# Characterizing Patients and Themes of SARS-CoV-2 Vaccine Hesitancy in a New York City High-Risk Older Population

**DOI:** 10.1093/geroni/igab046.2700

**Published:** 2021-12-17

**Authors:** Stephanie Chow, Yuji Yamada, Lizette Munoz, Susana Lavayen, Jodi Payne, Kavya Sreevalsan, Shamsi Fani

**Affiliations:** 1 Icahn School of Medicine at Mount Sinai, New York, New York, United States; 2 Icahn School of Medicine at Mount Sinai, Icahn School of Medicine at Mount Sinai, New York, United States; 3 Mount Sinai Health System, New York, New York, United States; 4 Mount Sinai Hospital, Mount Sinai Hospital, New York, United States

## Abstract

Background: Vaccines to prevent SARS-CoV-2 infection are deemed one of the most promising measures in controlling the devastating pandemic, yet there is significant vaccine hesitancy in some communities. Historic systemic health, discrimination, and structural inequities in specific racial and ethnic communities contribute to vaccine hesitancy with disproportionately negative impact. It is therefore critical to better understand vaccine hesitancy in this high-risk older population. The ALIGN (Acute Life Interventions, Goals, and Needs) program co-manages a panel of older patients with complex medical and psychosocial needs in an urban academic medical center. Methods: ALIGN enrolled or graduated Patients or designated healthcare proxies were contacted by telephone to discuss SARS-CoV-2 vaccine willingness and hesitancy using a standardized web-based survey. Qualitative data was categorized into themes and subgroups. Demographic data was collected by chart review. Results: Complete results are forthcoming and will include patient reported race and ethnicity baseline, vaccine hesitancy perceptions, with common overarching themes, and clinical team member debriefing. Iterative quality improvement actions taken based on elicited patient themes will also be included and assessed in telephone follow-up for changes in vaccine hesitancy. Conclusions: We are conducting a qualitative and quality improvement study characterizing vaccine perceptions and hesitancy in a high-risk older group with focus on racial and ethnic disparities in this population. This preliminary data informs healthcare providers of potential health literacy, cultural and language, and other potential barriers in order to help further understand how to optimize SARS-CoV-2 vaccine acceptance and delivery in a patient population with elevated risk.

